# Recent advances in gut microbiota-based nanotherapies for cancer treatment

**DOI:** 10.3389/fphar.2025.1617942

**Published:** 2025-12-04

**Authors:** Yuan Xu, Menglu Zhu, Yongchang Zhao, Enjia Chai, Miaolian Wu, Mengmeng Zhang

**Affiliations:** 1 Department of Pharmacy, the Fourth Affiliated Hospital of School of Medicine, and International School of Medicine, International Institutes of Medicine, Zhejiang University, Yiwu, Zhejiang, China; 2 Innovation Design Research, School of Innovation Design, China Academy of Art, Hangzhou, Zhejiang, China; 3 State Key Laboratory of Natural Medicines, Department of Pharmaceutics, China Pharmaceutical University, Nanjing, Jiangsu, China

**Keywords:** gut microbiota, nanotherapy, cancer, synergetic therapy, novel therapy

## Abstract

The effect of gut microbiota on carcinogenesis and progression has been demonstrated and arisen wide attention. Notably, specific microbial communities demonstrate dual therapeutic potential in enhancing anti-tumor treatment efficacy and mitigating chemotherapy-induced adverse effects, positioning microbial intervention as an innovative paradigm in oncology therapeutics. However, the clinical application of oral delivery of free-living gut microbiota faces significant challenges due to their inherent instability and premature clearance within the gastrointestinal tract. This biological limitation underscores the critical need for advanced delivery systems capable of preserving microbial viability and achieving targeted delivery. Nanotherapy, renowned for their precise targeting capabilities, exceptional biocompatibility, and enhanced pharmacokinetic profiles, have demonstrated transformative potential across biomedical applications, with several formulations already advancing to clinical implementation. Remarkably, current research on the delivery of gut microbiota for cancer treatment via nanotechnology remains limited and lacks comprehensive summarization. Therefore, this review systematically summarized recent advancements in gut microbiota-mediated nanotherapeutic strategies against cancer, proposing novel conceptual frameworks for synergistic microbiota-nanotherapy integration in cancer interventions.

## 1 Introduction

Cancer imposes a substantial burden on global healthcare systems and economic development. According to the latest data from the International Agency for Research on Cancer (IARC), approximately 20 million new cancer cases were diagnosed worldwide in 2022, with nearly 10 million deaths attributed to cancer related complications (Jokhadze et al., 2024). These statistics underscore the urgent need for innovative strategies to reduce the cancer burden and improve patient survival outcomes. Although traditional cancer therapies have made remarkable progress (Kaur et al., 2023), their clinical utility remains limited by suboptimal efficacy and significant side effects, highlighting the necessity for more effective therapeutic approaches (Chen et al., 2024).

In recent years, bacterial therapy has emerged as a promising modality in oncology, driven by advances in biomedical engineering. Certain bacterial species exhibit intrinsic tumor-targeting capabilities and are being explored for anticancer applications (Landa et al., 2025). Among these, gut microbiota, the complex microbial communities residing in the human gastrointestinal tract, comprise diverse microorganisms, including bacteria, archaea, eukaryotes, viruses, and parasites. Beyond their traditional role as passive modifiers of disease, gut microbiota actively influences carcinogenesis and treatment responses through modulation of drug absorption, distribution, metabolism, and excretion (ADME) (Kim, 2023; Zhao et al., 2023). Specific microbial taxa can impact tumor progression via three primary mechanisms: 1) metabolic reprogramming of the tumor microenvironment; 2) immunomodulation through regulation of innate and adaptive immunity; and 3) alteration of therapeutic responses to chemotherapy and immunotherapy (Lo et al., 2021). Consequently, modulation of the gut microbiota has been recognized as a novel strategy for cancer intervention (Chen Y. et al., 2021), with oral administration showing therapeutic potential in preclinical and clinical studies.

However, several challenges impede the clinical translation of gut microbiota-based therapies: 1) the dynamic nature of microbial communities may lead to unintended translocation and systemic immune activation, 2) poor targeting efficiency limits sufficient accumulation of beneficial microbes at tumor sites; and 3) the anti-tumor efficacy achieved by relying on the gut microbiota is insufficient (Feng et al., 2020; You et al., 2025). Therefore, developing spatiotemporally controlled delivery strategies is crucial to ensure both the safety and effectiveness of microbiota-based interventions.

**FIGURE 1:**
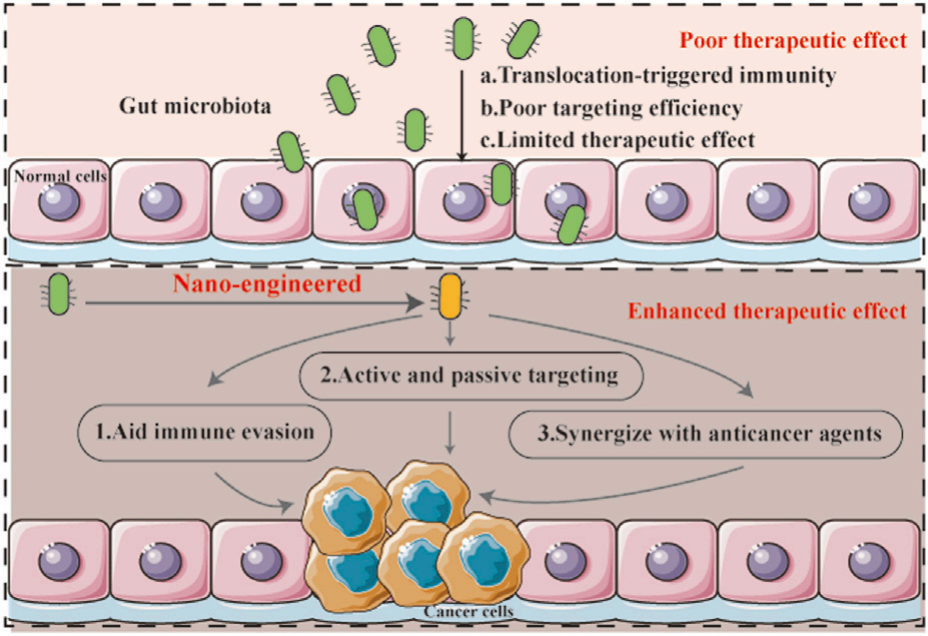
Mechanism schematic of nano-engineered gut microbiota for enhanced anti-cancer therapy.

Nanotherapy offers a promising solution to these limitations (Figure 1). 1) Nanocarriers can encapsulate live bacteria or microbial components, shielding them from immune recognition and preventing nonspecific tissue invasion. 2) Surface functionalization of nanoparticles with tumor-targeting ligands and combined with the enhanced permeability and retention (EPR) effect enables selective accumulation in tumor tissues. 3) Nano formulations can co-deliver microbiota-derived agents and therapeutic payloads (e.g., drugs or immunomodulators), enabling synergistic interactions between the microbiota and encapsulated therapeutics to enhance antitumor activity (Chehelgerdi et al., 2023; Niu et al., 2025).

In this review, we summarize recent advances in nano-enabled gut microbiota modulation for cancer therapy. We begin by outlining the functional roles of gut microbiota, followed by an overview of nanocarrier systems employed in tumor targeting. We then critically examine the current limitations of direct microbiota administration and highlight representative studies that integrate nanotechnology with microbiota-based interventions, illustrating the translational opportunities in oncology. By analyzing these developments, we aim to provide strategic insights for designing next-generation nanopharmaceuticals that harness the therapeutic potential of the gut microbiota while mitigating associated risks. This review seeks to bridge existing knowledge gaps and inspire innovation in personalized cancer nanomedicine.

## 2 Gut microbiota in cancer therapy

Gut microbiota including more than 2000 different bacterial strains, of which produces thousands of metabolites. They could critically regulate essential host physiological functions including nutrition metabolism, drug metabolism, intestinal barrier maintenance, immune homeostasis, and pathogen resistance (Almeida et al., 2019). The gut microbiota plays a crucial and multifaceted role in modulating the efficacy and toxicity of various cancer therapies, including chemotherapy, immunotherapy, and radiotherapy. Its influence is primarily mediated through immunomodulation, microbial metabolism, and maintenance of intestinal barrier integrity (Gao et al., 2025; Guo et al., 2020; He et al., 2024). Specific gut microbiota can directly metabolize chemotherapeutic drugs, activating or inactivating them, thereby influencing treatment outcomes and side effects like diarrhea and mucositis (Lang et al., 2023; Wu et al., 2025). In immunotherapy, particularly with immune checkpoint inhibitors (ICIs), commensals such as *Akkermansia muciniphila* and *Bifidobacterium spp*. enhance anti-tumor immunity by promoting the infiltration and activation of cytotoxic T-cells within the tumor microenvironment (Bolte et al., 2025). Conversely, dysbiosis often induced by the therapies themselves can lead to resistance and adverse events. Therapeutic strategies like probiotics, prebiotics, postbiotics, and fecal microbiota transplantation (FMT) are being explored to favorably reshape the microbial community, aiming to augment therapeutic responses and mitigate toxicities (Nobels et al., 2025). This burgeoning field highlights the gut microbiota as a pivotal determinant and a promising target for personalizing cancer treatment.

## 3 The advancements of nanotherapy

Compared to conventional drug delivery systems, nanomedicines exhibit superior therapeutic profiles, mainly attributable to their unique size, shape, and material composition, which enable targeted and precise drug delivery, improved pharmacokinetics and pharmacodynamics, and reduced systemic toxicity (Lammers and Ferrari, 2020). The most widely studied nanocarrier platforms include polymeric nanoparticles, liposomes, and inorganic nanoparticles (Zhou et al., 2022), each offering distinct advantages for specific therapeutic applications. Polymeric nanoparticles, made from biodegradable polymers, provide high drug loading, controlled release, and enhanced stability (Karati et al., 2024; Pontes and Grenha, 2020). Liposomes are spherical phospholipid vesicles that can encapsulate both hydrophilic and hydrophobic drugs, offering excellent biocompatibility (Dymek and Sikora, 2022). While numerous liposomal formulations have achieved clinical approval, current clinically approved liposome-based drugs rely primarily on passive targeting via the enhanced permeability and retention (EPR) effect, which suffers from low targeting efficiency and inter-patient variability. To overcome this limitation, active targeting strategies by employing ligands such as antibodies, peptides, or aptamers for molecular recognition, are being actively explored to direct liposomes to specific cell types or tissues (Guan et al., 2018). Inorganic nanomedicines utilize inorganic materials as carriers, such as mesoporous silica or gold nanoparticles. Compared to organic counterparts (e.g., polymeric nanoparticles and liposomes), it offers greater precision in tuning physicochemical properties, enabling advanced functionalities like imaging, stimuli-responsive release, and combination therapy (Molkenova et al., 2022). In addition to these major categories, other emerging platforms, such as protein-based nanoparticles and nanogels, are also playing increasingly important roles in drug delivery, offering unique advantages in biocompatibility, responsiveness, and multifunctionality (Dan et al., 2025; Liu Y. et al., 2025).

## 4 The application of gut microbiota combined nanotherapy for cancer treatment

With a deeper understanding of the gut microbiota, increasing evidence has highlighted its critical role in the initiation and progression of cancer (Fernandes et al., 2022). Notably, certain microbial species, classified as pro-carcinogenic, anti-carcinogenic and their bioactive metabolites provide a strong foundation for microbiota-targeted interventions. Specific gut microbiota and their metabolites can be leveraged to enhance anti-tumor efficacy through their intrinsic anti-tumor activity, natural tumor-targeting capacity, and ability to synergize with co-administered therapeutics (Chen Z. X. et al., 2021; Yang et al., 2021; Ye et al., 2024). However, conventional oral microbiota-based formulations face several challenges. To overcome these limitations, nanotechnology has emerged as a powerful strategy for the targeted and controlled delivery of both microbiota-derived agents and therapeutic drugs. This has led to the emergence of a new class of microbiota-based cancer nanotherapeutics. Based on their primary mode of action and combinatorial focus, we categorize these approaches into three strategic directions: combination with (i) conventional anticancer agents, (ii) immunotherapies, and (iii) emerging modalities such as photodynamic or sonodynamic therapy (Table 1).

**TABLE 1:** A comparative summary of nanodelivery systems utilizing microbiota and metabolites for cancer therapy.

Type	Microbiota type	Nanocarrier	Treated disease	Mechanism	References
Combined with anticancer agents	*Lactobacillus*	Extracellular polymeric substances extracted from a marine bacterium P. *aeruginosa* JP-11	Breast cancer	Anaerobic bacteria target the hypoxic region of tumor and the effect of anticancer agents	Raj and Das (2017)
	*Lactobacillus*	Nano-engineered bacteria	Nasopharyngeal carcinoma	Anaerobic bacteria target the hypoxic region of tumor and the effect of anticancer agents	Shen et al. (2024)
Combined with immunotherapy	Microbial metabolites: Short-chain fatty acids	Prebiotic xylan and stearic acid conjugate	Colorectal cancer	Strengthen the anti-tumor immune response	Lang et al. (2023)
	Microbial metabolites: Butyric acid	mPEG-PLGA-PLL	Hepatocellular carcinoma	Strengthen the anti-tumor immune response	Che et al. (2023)
	*Escherichia coli Nissle1917*	Supraparticles made from green tea polyphenols and milk protein	Colorectal cancer	Reprogramd the tumor microenvironment and enhance systemic immune responses	Liu Q. et al. (2025)
Combined with emerging modalities	*Bifidobacterium bifidum*	Nano-engineered bacteria	Colorectal cancer	Photothermal therapy and enhanced immune response	Reghu and Miyako (2022)
	*Escherichia coli*	Nano-engineered bacteria	Colorectal cancer	Photodynamic therapy and the effect of perforin	Tao et al. (2023)
	*Salmonella typhimurium strain*	Nano-engineered bacteria	Opaque melanoma	Photodynamic therapy and enhanced immune response	Yang et al. (2022)
	*Bifidobacterium*	Nano-engineered bacteria	Laryngocarcinoma	Tumor-targeted photodynamic and sonodynamic synergistic therapy	Li et al. (2022)

### 4.1 Gut microbiota combing with anticancer agents

The targeted delivery of gut microbiota or their bioactive metabolites in combination with anticancer agents via nanotechnology holds significant therapeutic potential (Damoogh et al., 2021). This innovative strategy synergizes the precision of nanocarriers with the growing understanding of the gut microbiome’s role in cancer biology, offering a promising avenue to enhance treatment efficacy while minimizing systemic toxicity. Notably, the tumor microenvironment, which characterized by hypoxia, nutrient abundance, and immunosuppression, creates favorable conditions for the selective enrichment of anaerobic or facultative anaerobic bacteria. This intrinsic tropism enables certain microbial species to serve as natural tumor-targeting vectors (Nguyen and Min, 2017). For instance, Raj et al. engineered facultative anaerobic lactic acid bacteria to carry fluorescent cadmium sulfide nanoparticles for targeting human breast cancer cells. Confocal laser scanning microscopy and field emission scanning electron microscopy revealed successful encapsulation of cadmium sulfide by *Lactobacillus* and its effective delivery to tumor sites. These findings suggested that specific gut microbiota can function as biocompatible, tumor-homing vehicles for the targeted delivery of anticancer agents (Raj and Das, 2017).

Based on this concept, Shen et al. developed a targeted drug delivery system using genetically engineered *Lactobacillus* plantarum to deliver the prodrug 7-ethyl-10-hydroxycamptothecin (SN38). The engineered strain expressed a surface-displayed oligopeptide-binding protein that specifically interacts with heparan sulfate proteoglycans overexpressed on tumor cells surfaces. This modification conferred strong binding affinity to multiple cancer types, including nasopharyngeal, bladder, lung, and gastric carcinomas. In a male Balb/c nude mouse xenograft model, this targeted approach significantly enhanced the antitumor efficacy of SN38, resulting in a 54% greater tumor growth inhibition compared to controls (Shen et al., 2024).

### 4.2 Gut microbiota combing with immunotherapy

With the deepening understanding of the role of gut microbiota in cancer therapy, its potential to modulate immune responses has garnered widespread attention (Yi et al., 2019). Some microorganisms such as *Lactobacillus*, *Bacteroides fragilis*, *Eubacterium limosum* and *Faecalibacterium spp*. have been proved to be able to increase the sensitivity of tumors to immunotherapy and enhance anti-tumor efficacy (Masheghati et al., 2024; Rastogi and Singh, 2022). For example, Liu et al. used *Lactobacillus* as a carrier to deliver selenium and designed a selenium-rich *Lactobacillus* brevis nano-system (SeL). Compared with heat-inactivated nano selenium-enriched *Lactobacillus* brevis (HiSeL), study showed that SeL could enhance the effect of alum adjuvant on improving vaccine (inactivated *Clostridium perfringens* type A vaccine) immunogenicity by inducing antibody production more quickly, obtaining higher immunoglobulin G antibody level, improving secretory immunoglobulin A antibody level, enhancing cellular immune response and regulating Th1/Th2 immune response (Liu et al., 2023).

In addition to their direct tumor-targeting potential, gut microbiota can exert anti-tumor effects through their bioactive metabolites. For example, He et al. demonstrated that microbial metabolites, particularly butyrate, can upregulate the expression of inhibitor of DNA binding 2 (ID2) in CD8^+^ T cells, thereby enhancing their cytotoxic activity and boosting anti-tumor immune responses (He et al., 2021). Based on this mechanism, Lang et al. developed a nano-delivery platform SCXN, a capecitabine-loaded nanoparticle using the prebiotic xylan-stearic acid conjugate. The system leveraged xylan as a prebiotic to selectively promote the growth of beneficial gut bacteria and stimulate the production of short-chain fatty acids (SCFAs), particularly butyrate. In murine models of colorectal cancer, SCXN significantly enhanced anti-tumor immunity, increasing the tumor growth inhibition rate from 5.29% (control) to 71.78%, and extending median survival from 14 to 33.5 days. This study represented a successful integration of chemotherapy and microbiota modulation through nanotechnology. By harnessing the immunomodulatory properties of microbiota-derived butyrate, the platform not only improved chemotherapeutic efficacy but also reshaped the anti-tumor immune microenvironment, offering a promising combinatorial strategy for cancer therapy (Lang et al., 2023).

Additionally, Che et al. developed a targeted nanoparticle system for the co-delivery of the intestinal microbial metabolite butyric acid and the chemotherapeutic agent sorafenib. The nanoparticles were formulated using a mPEG-PLGA-PLL (Methoxy Poly (ethylene glycol)-Poly (lactic-co-glycolic acid)-Poly (L-lysine)) copolymer and surface-functionalized with an anti-Glypican-3 antibody to enable tumor-specific targeting. This engineered platform significantly prolonged drug retention time, reduced systemic toxicity, and increased drug accumulation at the tumor site. Notably, butyrate was shown to potentiate anti-tumor immunity by activating and recruiting CD8^+^ T cells. Its combination with sorafenib yielded a synergistic effect, leading to significantly improved therapeutic outcomes. Overall, this system exhibited potent anti-tumor activity in preclinical models, highlighting the therapeutic potential of integrating microbiota-derived metabolites with targeted chemotherapy via nanotechnology (Che et al., 2023). Liu et al. engineered beneficial bacteria by coating *Escherichia coli* Nissle1917 with supraparticles made from green tea polyphenols and milk protein. This “armor” protected the bacteria from chemotherapy through non-covalent interactions, boosting their survival by 27-fold. The protected bacteria (nanoarmored live bacterial biotherapeutics, supraLBT) restored a healthy gut microbiome and immune balance, which reprogramd the tumor microenvironment. This led to fewer immunosuppressive T cells and more tumor-infiltrating cancer-killing CD8^+^ T cells. Consequently, combining oral supraLBT with doxorubicin chemotherapy resulted in a 2.35-fold greater tumor regression than chemotherapy alone, demonstrating a powerful synergy (Liu Q. et al., 2025).

### 4.3 Gut microbiota combing with emerging modalities

In recent years, emerging therapeutic modalities such as photothermal therapy (PTT) and photodynamic therapy (PDT) that utilize bacteria as delivery vectors have demonstrated significant potential in cancer treatment. These approaches not only enable localized tumor ablation but also hold promise for triggering robust anti-tumor immune responses, thereby enhancing overall therapeutic efficacy. For instance, Reghu et al. developed a novel nanoplatform using *Bifidobacterium bifidum* (BB) as a living vector. The system was engineered by encapsulating the organic photosensitizer indocyanine green (ICG) with Cremophor EL (CRE) through a simple incubation and washing protocol, preserving both bacterial morphology and viability. The functionalized bacteria exhibited strong near-infrared (NIR) absorbance, distinct fluorescence, high photothermal conversion efficiency, low toxicity, and inherent tumor-targeting capability due to the anaerobic nature of BB. Upon exposure to biocompatible NIR laser irradiation, the engineered bacteria generated significant photothermal effects and an enhanced local immune response, leading to rapid elimination of colorectal tumors in mice (Reghu and Miyako, 2022). Furthermore, Tao et al. constructed genetically modified *E. coli* MG1655 to generate HlyE perforin, which coated with lanthanide-doped upconversion nanoparticles (UCNPs). Leveraging the innate chemotactic preference of *E. coli* for hypoxic tumor microenvironments, the system achieved efficient tumor accumulation. Upon NIR irradiation, the UCNPs emitted visible light that activated the genetically encoded toxin, HlyE perforin, inducing targeted cancer cell lysis. This innovative strategy not only enables precise spatiotemporal control of therapy but also offers a promising solution to the challenge of tumor hypoxia (Tao et al., 2023).

Additionally, Yang et al. used *Salmonella typhimurium* strain to design engineered luminescent bacteria which could synergistically enhance PDT and anti-tumor immunity efficacy. Through using firefly-luciferase-expressing plasmid (Luc-S.T.ΔppGpp) as an internal light source, attenuated *S. typhimurium* strain ΔppGpp (S.T.ΔppGpp) was transformed to design bioluminescent bacteria to uniformly illuminate the whole tumor. After the formation of hydrogel in the tumor, the implanted Luc-S.T.ΔppGpp and D-fluorescein would continuously generate light to excite the photosensitizer chlorin e6 (Ce6), which could effectively inhibit different types of tumors. And the enhanced PDT of Luc-S.T.ΔppGpp could also trigger effective anti-tumor immunity to inhibit tumor metastasis. This bio-triggered PDT made up for the limited penetration depth of traditional PDT and was expected to be used for potential clinical transformation (Yang et al., 2022).

Furthermore, the combined application of various novel treatment approaches has also garnered significant attention. Li et al. designed a tumor-targeted photodynamic and sonodynamic synergistic therapy system based on *Bifidobacterium*. In this delivery system, Ce6 nanoparticles were prepared, then they were mixed into *Bifidobacterium* and conjugated with anti-death receptor 5 antibody (anti-DR5 Ab). ROS generation triggered by concurrent 671 nm laser and ultrasound irradiation yielded a superior tumor-suppressive effect compared to individual modalities, demonstrating a distinct synergistic anti-tumor action. Moreover, the *Bifidobacterium*-based vector was shown to be gradually cleared *in vivo*, exhibiting favorable biocompatibility and biosafety. These features, combined with its intrinsic tumor-targeting capability, made this bacterial system a promising platform for targeted cancer therapy and offered new opportunities for the development of microbiota-inspired theranostic strategies (Li et al., 2022). Overall, the integration of novel technologies has empowered gut microbiota and nanotherapy to exert increasingly precise and potent roles in anticancer therapeutics, representing a critical frontier for future research.

## 5 Conclusion and perspectives

The integration of gut microbiota with nanotherapy represents a paradigm shift in oncology, leveraging microbial immunomodulatory and metabolic capabilities to overcome biological barriers and enhance drug efficacy. In the realm of personalized microbiota-nanotherapy, the future lies in leveraging artificial intelligence and multi-omics data to delineate patient-specific microbial signatures. This will enable the rational design of bespoke nano-formulations, where CRISPR (Clustered Regularly Interspaced Short Palindromic Repeats)-edited probiotics or synthetic microbial consortia are tailored to an individual’s microbiome profile and tumor microenvironment, ushering in a new era of pharmacomicrobiomics. However, these technological advancements are accompanied by profound ethical and translational considerations. Biosafety remains a paramount concern, particularly regarding the long-term ecological impact and evolutionary stability of genetically modified microbes within the human gut ecosystem. Rigorous preclinical assessment is required to evaluate potential off-target effects, horizontal gene transfer risks, and unintended microbiome dysbiosis. Moreover, the regulatory framework for these living therapeutics remains largely undefined. Their hybrid nature, as a combination of drug, biologic, and live microorganism, poses unique classification and approval challenges that current regulatory paradigms are ill-equipped to address. The application of gut microbiota with nanotherapy has now not been studied in human and needs further consideration.

Ultimately, realizing the full clinical potential of microbiota-nanoparticle co-delivery systems will depend on sustained cross-disciplinary collaboration among microbiologists, nanotechnologists, immunologists, clinicians, bioethicists, and regulatory experts. Only through such integrated efforts can these sophisticated platforms evolve from promising preclinical concepts into mainstream oncological interventions.
